# Supplementary material for: A Likelihood Approach for Real-Time Calibration of Stochastic Compartmental Epidemic Models
Source: PLoS Comput Biol. 2017 Jan 17;13(1):e1005257. doi: 10.1371/journal.pcbi.1005257 (PMC5240920; doi:10.1371/journal.pcbi.1005257)
Supplement: S1 File — (TAR.GZ) [file pcbi.1005257.s014.tar.gz › HSPH_Online-SI-Revision/EnKF07/n1-extreme/n1-extreme_EnKF2_table-8.pdf]

1	newly 3 weeks spec	6.	42.5	{1., 105.}	636.333	0.79565	newly 3 weeks cumul	8.	318.95	{19., 835.}	3895.13	1.35283	newly 5 weeks spec	31.	28.19	{0., 91.}	108.871	1.16544	newly 5 weeks cumul	58.	381.46	{24., 836.}	576.517	0.757016	newly 68 weeks cumul 923. 496.94 {38., 907.} 47.6056 0.531363
2	newly 3 weeks spec	47.	50.85	{3., 121.}	74.9574	0.472327	newly 3 weeks cumul	87.	368.83	{148., 865.}	327.805	0.549356	newly 5 weeks spec	79.	17.66	{0., 45.}	79.8228	1.26373	newly 5 weeks cumul	238.	417.62	{167., 990.}	93.3782	0.26249	newly 64 weeks cumul 911. 445.95 {171., 1037.} 56.899 0.435444
3	newly 3 weeks spec	17.	87.51	{6., 220.}	435.824	0.679885	newly 3 weeks cumul	33.	496.19	{152., 901.}	1409.06	1.08564	newly 5 weeks spec	43.	36.98	{0., 87.}	71.3953	0.758172	newly 5 weeks cumul	103.	587.81	{225., 1059.}	478.515	0.72003	newly 66 weeks cumul 907. 671.98 {274., 1119.} 40.8556 0.291769
4	newly 3 weeks spec	11.	57.01	{7., 125.}	434.818	0.648341	newly 3 weeks cumul	27.	520.22	{107., 1001.}	1826.74	1.16716	newly 5 weeks spec	51.	17.5	{0., 47.}	77.4902	1.25196	newly 5 weeks cumul	107.	568.02	{124., 1060.}	435.402	0.651416	newly 65 weeks cumul 788. 603.78 {143., 1092.} 45.1371 0.292178
5	newly 3 weeks spec	48.	7.07	{0., 17.}	88.2708	1.69264	newly 3 weeks cumul	91.	68.39	{5., 80.}	90.7143	0.608423	newly 5 weeks spec	68.	2.94	{0., 8.}	95.6765	2.27261	newly 5 weeks cumul	214.	75.31	{5., 93.}	87.9953	0.89839	newly 58 weeks cumul 910. 80.79 {5., 104.} 91.6033 1.46009
6	newly 3 weeks spec	34.	39.79	{0., 75.}	92.1471	0.576477	newly 3 weeks cumul	108.	309.54	{78., 703.}	199.222	0.40291	newly 5 weeks spec	27.	11.84	{0., 32.}	84.8148	1.1449	newly 5 weeks cumul	166.	343.11	{96., 703.}	127.669	0.318535	newly 63 weeks cumul 696. 361.87 {108., 857.} 65.3664 0.499705
7	newly 3 weeks spec	32.	89.86	{3., 188.}	218.188	0.631202	newly 3 weeks cumul	67.	430.8	{86., 829.}	552.239	0.745666	newly 5 weeks spec	59.	46.62	{0., 117.}	63.322	0.728335	newly 5 weeks cumul	173.	544.3	{131., 927.}	228.728	0.503018	newly 67 weeks cumul 867. 662.05 {216., 1051.} 34.5502 0.246813
8	newly 3 weeks spec	33.	10.27	{0., 19.}	84.4545	1.12037	newly 3 weeks cumul	77.	132.53	{18., 264.}	117.571	0.364534	newly 5 weeks spec	70.	2.81	{0., 5.}	95.9857	2.12442	newly 5 weeks cumul	203.	140.89	{18., 345.}	75.7094	0.525279	newly 61 weeks cumul 775. 145.16 {18., 407.} 82.7561 0.998544
9	newly 3 weeks spec	10.	47.46	{2., 120.}	406.2	0.700184	newly 3 weeks cumul	20.	307.09	{20., 794.}	1445.95	0.980065	newly 5 weeks spec	41.	33.75	{0., 105.}	96.9512	1.13226	newly 5 weeks cumul	80.	379.89	{29., 794.}	396.263	0.622821	newly 68 weeks cumul 770. 518.01 {58., 934.} 42.0792 0.409269
10	newly 3 weeks spec	30.	10.05	{0., 20.}	81.1	1.03993	newly 3 weeks cumul	68.	166.6	{17., 439.}	178.294	0.408796	newly 5 weeks spec	62.	1.96	{0., 3.}	96.9677	2.28756	newly 5 weeks cumul	180.	173.19	{17., 444.}	79.8056	0.43665	newly 61 weeks cumul 869. 175.68 {17., 444.} 79.7837 0.939831
11	newly 3 weeks spec	30.	86.29	{3., 192.}	226.3	0.628892	newly 3 weeks cumul	41.	440.29	{91., 871.}	978.61	0.927855	newly 5 weeks spec	78.	44.84	{0., 118.}	62.6667	0.824911	newly 5 weeks cumul	165.	548.19	{124., 952.}	244.988	0.507678	newly 67 weeks cumul 767. 660.56 {224., 1070.} 35.8305 0.22506
12	newly 3 weeks spec	6.	42.5	{1., 105.}	636.333	0.79565	newly 3 weeks cumul	12.	318.95	{19., 835.}	2567.58	1.19843	newly 5 weeks spec	33.	28.19	{0., 91.}	104.939	1.17739	newly 5 weeks cumul	59.	381.46	{24., 836.}	565.593	0.751967	newly 68 weeks cumul 724. 496.94 {38., 907.} 44.7459 0.47119
13	newly 3 weeks spec	17.	10.29	{0., 18.}	79.4706	0.861403	newly 3 weeks cumul	47.	149.49	{17., 411.}	246.064	0.456879	newly 5 weeks spec	37.	2.43	{0., 4.}	93.7568	1.92338	newly 5 weeks cumul	107.	156.49	{17., 439.}	100.813	0.366536	newly 61 weeks cumul 729. 160.02 {17., 439.} 79.9506 0.932262
14	newly 3 weeks spec	25.	61.29	{2., 130.}	193.8	0.606176	newly 3 weeks cumul	67.	552.11	{189., 1034.}	729.478	0.838464	newly 5 weeks spec	83.	19.53	{0., 53.}	79.7229	1.42216	newly 5 weeks cumul	206.	604.72	{229., 1163.}	204.583	0.458882	newly 65 weeks cumul 782. 640.81 {229., 1182.} 44.9604 0.286346
15	newly 3 weeks spec	26.	18.87	{0., 43.}	108.885	1.02773	newly 3 weeks cumul	51.	158.93	{18., 547.}	242.412	0.368487	newly 5 weeks spec	70.	6.88	{0., 16.}	91.1429	1.96674	newly 5 weeks cumul	147.	176.68	{18., 547.}	117.782	0.524839	newly 60 weeks cumul 692. 189.69 {19., 610.} 81.5275 0.977604
16	newly 3 weeks spec	24.	84.88	{3., 192.}	292.167	0.682497	newly 3 weeks cumul	40.	446.43	{96., 917.}	1020.38	0.952144	newly 5 weeks spec	42.	44.2	{0., 118.}	84.4286	0.796527	newly 5 weeks cumul	120.	553.11	{117., 968.}	370.108	0.636079	newly 67 weeks cumul 755. 657.95 {201., 1051.} 36.7854 0.251359
17	newly 3 weeks spec	26.	53.83	{4., 123.}	149.269	0.502697	newly 3 weeks cumul	47.	379.83	{124., 838.}	709.894	0.813824	newly 5 weeks spec	50.	17.66	{0., 45.}	72.8	1.06232	newly 5 weeks cumul	121.	427.11	{157., 933.}	257.215	0.481563	newly 64 weeks cumul 819. 452.25 {162., 991.} 53.1856 0.382294
18	newly 3 weeks spec	13.	84.29	{1., 195.}	574.692	0.836761	newly 3 weeks cumul	23.	422.28	{58., 837.}	1740.17	1.14552	newly 5 weeks spec	42.	44.49	{0., 115.}	82.5476	0.744501	newly 5 weeks cumul	99.	529.39	{114., 932.}	443.283	0.675298	newly 67 weeks cumul 912. 643.29 {162., 1040.} 35.9331 0.275265
19	newly 3 weeks spec	14.	43.67	{1., 115.}	257.929	0.653665	newly 3 weeks cumul	34.	308.32	{19., 818.}	821.118	0.783448	newly 5 weeks spec	39.	29.8	{0., 99.}	97.9487	1.21037	newly 5 weeks cumul	99.	372.07	{42., 826.}	302.576	0.574017	newly 68 weeks cumul 880. 500.99 {42., 916.} 45.8739 0.497137
20	newly 3 weeks spec	50.	12.51	{0., 22.}	86.7	1.27096	newly 3 weeks cumul	121.	158.	{27., 349.}	88.9091	0.336006	newly 5 weeks spec	81.	3.83	{0., 6.}	95.2716	2.25615	newly 5 weeks cumul	273.	168.14	{27., 407.}	76.0366	0.543536	newly 61 weeks cumul 893. 173.12 {27., 438.} 82.4278 0.962778
21	newly 3 weeks spec	10.	52.91	{3., 132.}	453.1	0.720121	newly 3 weeks cumul	39.	383.45	{141., 875.}	887.615	0.917622	newly 5 weeks spec	27.	18.29	{0., 40.}	73.1481	0.859337	newly 5 weeks cumul	84.	432.52	{159., 969.}	420.405	0.661937	newly 64 weeks cumul 711. 457.15 {164., 1037.} 52.3952 0.386047
22	newly 3 weeks spec	50.	53.82	{5., 121.}	72.56	0.424711	newly 3 weeks cumul	92.	526.74	{170., 977.}	477.261	0.675268	newly 5 weeks spec	80.	16.63	{0., 44.}	81.9375	1.47024	newly 5 weeks cumul	222.	571.6	{234., 1066.}	168.577	0.393824	newly 65 weeks cumul 825. 606.81 {234., 1075.} 43.3636 0.28811
23	newly 3 weeks spec	21.	83.77	{2., 188.}	333.476	0.695792	newly 3 weeks cumul	38.	445.28	{97., 903.}	1075.63	0.95944	newly 5 weeks spec	50.	45.08	{0., 117.}	75.68	0.814904	newly 5 weeks cumul	115.	553.59	{116., 965.}	390.617	0.636945	newly 67 weeks cumul 744. 660.12 {185., 1051.} 36.4597 0.22702
24	newly 3 weeks spec	11.	42.5	{1., 105.}	323.636	0.657254	newly 3 weeks cumul	16.	318.95	{19., 835.}	1904.44	1.09214	newly 5 weeks spec	41.	28.19	{0., 91.}	93.0488	1.21887	newly 5 weeks cumul	85.	381.46	{24., 836.}	373.624	0.653019	newly 68 weeks cumul 886. 496.94 {38., 907.} 46.4131 0.517838
25	newly 3 weeks spec	21.	54.96	{4., 110.}	191.143	0.492063	newly 3 weeks cumul	34.	525.72	{173., 973.}	1447.	1.08363	newly 5 weeks spec	31.	18.04	{0., 49.}	79.4194	0.996891	newly 5 weeks cumul	93.	573.55	{231., 1025.}	520.806	0.722234	newly 65 weeks cumul 758. 610.63 {233., 1065.} 42.8298 0.263994
26	newly 3 weeks spec	12.	86.59	{2., 196.}	646.917	0.870168	newly 3 weeks cumul	15.	442.74	{103., 881.}	2854.53	1.36013	newly 5 weeks spec	48.	44.51	{0., 116.}	75.0208	0.767148	newly 5 weeks cumul	84.	550.36	{128., 968.}	561.095	0.765732	newly 67 weeks cumul 923. 660.89 {169., 1074.} 35.2145 0.273674
27	newly 3 weeks spec	38.	32.1	{1., 65.}	87.2105	0.653763	newly 3 weeks cumul	98.	213.85	{29., 473.}	156.561	0.394847	newly 5 weeks spec	56.	10.51	{0., 28.}	86.625	1.4935	newly 5 weeks cumul	192.	241.64	{29., 623.}	92.9167	0.406803	newly 62 weeks cumul 795. 268.44 {29., 681.} 74.7447 0.786472
28	newly 3 weeks spec	39.	98.11	{11., 212.}	177.359	0.463649	newly 3 weeks cumul	74.	489.45	{158., 885.}	569.392	0.769408	newly 5 weeks spec	88.	43.93	{0., 90.}	58.4886	0.746042	newly 5 weeks cumul	201.	597.77	{232., 1045.}	206.871	0.471521	newly 66 weeks cumul 916. 698.6 {284., 1140.} 38.7926 0.272464
29	newly 3 weeks spec	8.	44.42	{3., 81.}	480.	0.662332	newly 3 weeks cumul	18.	314.46	{80., 802.}	1650.67	1.08881	newly 5 weeks spec	61.	14.07	{0., 37.}	83.6885	1.37624	newly 5 weeks cumul	116.	352.18	{94., 868.}	216.914	0.42721	newly 63 weeks cumul 930. 374.71 {101., 891.} 68.4656 0.60359
30	newly 3 weeks spec	51.	9.24	{0., 21.}	89.0588	1.68658	newly 3 weeks cumul	155.	117.08	{9., 296.}	106.632	0.713987	newly 5 weeks spec	44.	2.61	{0., 5.}	94.75	2.04378	newly 5 weeks cumul	251.	124.79	{9., 316.}	95.9402	0.848759	newly 58 weeks cumul 605. 129.56 {9., 321.} 87.5207 1.14455
31	newly 3 weeks spec	24.	86.59	{2., 196.}	294.208	0.6906	newly 3 weeks cumul	52.	442.74	{103., 881.}	756.769	0.850055	newly 5 weeks spec	60.	44.51	{0., 116.}	65.45	0.792953	newly 5 weeks cumul	138.	550.36	{128., 968.}	308.696	0.584044	newly 67 weeks cumul 874. 660.89 {169., 1074.} 35.1934 0.265256
32	newly 3 weeks spec	21.	88.08	{2., 192.}	355.619	0.745588	newly 3 weeks cumul	42.	435.74	{51., 846.}	943.048	0.9113	newly 5 weeks spec	41.	44.1	{0., 117.}	89.2683	0.815648	newly 5 weeks cumul	113.	543.72	{83., 913.}	393.274	0.649806	newly 67 weeks cumul 845. 648.78 {153., 1033.} 34.7006 0.270921
33	newly 3 weeks spec	14.	44.33	{3., 88.}	252.5	0.535085	newly 3 weeks cumul	41.	306.67	{81., 792.}	653.049	0.74948	newly 5 weeks spec	35.	15.76	{0., 40.}	84.	1.08521	newly 5 weeks cumul	96.	347.34	{97., 940.}	269.896	0.466699	newly 63 weeks cumul 860. 374.02 {112., 978.} 68.2674 0.568861
34	newly 3 weeks spec	20.	92.09	{6., 208.}	382.65	0.644073	newly 3 weeks cumul	48.	505.75	{161., 956.}	960.188	0.939117	newly 5 weeks spec	70.	40.1	{0., 88.}	60.5429	0.813568	newly 5 weeks cumul	151.	606.3	{233., 1057.}	311.099	0.583827	newly 66 weeks cumul 845. 692.34 {282., 1174.} 42.0923 0.287266
35	newly 3 weeks spec	18.	86.01	{3., 194.}	409.389	0.728266	newly 3 weeks cumul	50.	451.54	{94., 965.}	809.4	0.875948	newly 5 weeks spec	58.	44.03	{0., 117.}	69.3621	0.901626	newly 5 weeks cumul	143.	558.93	{117., 977.}	303.294	0.59082	newly 67 weeks cumul 834. 662.35 {145., 1058.} 35.9269 0.2755
36	newly 3 weeks spec	24.	54.92	{4., 122.}	167.083	0.522359	newly 3 weeks cumul	59.	381.83	{131., 862.}	550.051	0.721189	newly 5 weeks spec	53.	17.33	{0., 41.}	72.0189	1.07553	newly 5 weeks cumul	146.	429.72	{162., 999.}	199.808	0.412879	newly 64 weeks cumul 848. 455.66 {169., 1002.} 54.1651 0.401227
37	newly 3 weeks spec	45.	19.58	{0., 29.}	102.311	1.19502	newly 3 weeks cumul	72.	128.82	{13., 288.}	165.028	0.465589	newly 5 weeks spec	59.	5.17	{0., 12.}	91.5085	1.88222	newly 5 weeks cumul	179.	144.06	{14., 425.}	113.821	0.717099	newly 59 weeks cumul 848. 152.43 {14., 454.} 86.8078 1.22666
38	newly 3 weeks spec	27.	52.26	{6., 123.}	136.37	0.465088	newly 3 weeks cumul	70.	366.82	{129., 858.}	427.2	0.635917	newly 5 weeks spec	62.	17.97	{0., 42.}	77.4032	1.18315	newly 5 weeks cumul	186.	414.82	{154., 1006.}	133.559	0.322653	newly 64 weeks cumul 878. 442.88 {156., 1030.} 56.6014 0.431046
39	newly 3 weeks spec	9.	86.01	{3., 194.}	879.667	0.91237	newly 3 weeks cumul	17.	451.54	{94., 965.}	2558.82	1.31059	newly 5 weeks spec	28.	44.03	{0., 117.}	130.821	0.871335	newly 5 weeks cumul	66.	558.93	{117., 977.}	752.803	0.869025	newly 67 weeks cumul 893. 662.35 {145., 1058.} 35.4546 0.284038
40	newly 3 weeks spec	58.	55.02	{4., 122.}	67.1379	0.452203	newly 3 weeks cumul	138.	506.29	{117., 976.}	276.819	0.512987	newly 5 weeks spec	45.	18.55	{0., 43.}	79.2667	1.1825	newly 5 weeks cumul	245.	555.53	{128., 1073.}	140.829	0.355332	newly 65 weeks cumul 775. 597.7 {157., 1134.} 45.8323 0.291404
41	newly 3 weeks spec	31.	19.69	{0., 39.}	110.935	1.22412	newly 3 weeks cumul	62.	140.28	{9., 300.}	176.097	0.394729	newly 5 weeks spec	37.	6.12	{0., 18.}	90.1081	1.78825	newly 5 weeks cumul	130.	158.25	{9., 396.}	116.731	0.523446	newly 60 weeks cumul 655. 167.56 {9., 502.} 83.7191 1.02363
42	newly 3 weeks spec	20.	42.5	{1., 105.}	166.9	0.590699	newly 3 weeks cumul	35.	318.95	{19., 835.}	827.686	0.822197	newly 5 weeks spec	52.	28.19	{0., 91.}	83.3654	1.26748	newly 5 weeks cumul	108.	381.46	{24., 836.}	282.963	0.598732	newly 68 weeks cumul 918. 496.94 {38., 907.} 47.4292 0.529476
43	newly 3 weeks spec	9.	84.88	{3., 192.}	867.778	0.917081	newly 3 weeks cumul	17.	446.43	{96., 917.}	2529.12	1.30555	newly 5 weeks spec	64.	44.2	{0., 118.}	65.4375	0.847845	newly 5 weeks cumul	106.	553.11	{117., 968.}	429.953	0.68082	newly 67 weeks cumul 902. 657.95 {201., 1051.} 35.1031 0.2717
44	newly 3 weeks spec	40.	54.04	{5., 121.}	88.95	0.42828	newly 3 weeks cumul	67.	524.07	{165., 971.}	685.448	0.798863	newly 5 weeks spec	68.	15.98	{0., 47.}	80.5882	1.38281	newly 5 weeks cumul	179.	567.87	{238., 1007.}	226.665	0.468244	newly 65 weeks cumul 873. 603.33 {239., 1069.} 42.7755 0.297391
45	newly 3 weeks spec	11.	43.33	{1., 120.}	336.455	0.686635	newly 3 weeks cumul	17.	310.48	{19., 825.}	1736.94	1.04791	newly 5 weeks spec	50.	29.24	{0., 96.}	85.96	1.2909	newly 5 weeks cumul	90.	372.87	{24., 828.}	341.589	0.630047	newly 68 weeks cumul 926. 485.15 {35., 917.} 48.8737 0.557546
46	newly 3 weeks spec	19.	55.25	{6., 141.}	221.421	0.506808	newly 3 weeks cumul	34.	515.7	{112., 1011.}	1417.24	1.06004	newly 5 weeks spec	55.	17.	{0., 43.}	77.5273	1.25366	newly 5 weeks cumul	129.	562.35	{127., 1072.}	342.659	0.575287	newly 65 weeks cumul 889. 598.78 {149., 1101.} 44.3487 0.315137
47	newly 3 weeks spec	19.	88.	{8., 206.}	382.	0.639777	newly 3 weeks cumul	29.	481.58	{156., 891.}	1565.52	1.13531	newly 5 weeks spec	39.	37.44	{0., 87.}	73.1795	0.694977	newly 5 weeks cumul	94.	575.28	{235., 1049.}	518.383	0.752258	newly 66 weeks cumul 840. 657.69 {278., 1122.} 42.4798 0.299056
48	newly 3 weeks spec	17.	97.01	{8., 214.}	486.059	0.699443	newly 3 weeks cumul	48.	492.3	{148., 896.}	932.958	0.936407	newly 5 weeks spec	41.	43.52	{0., 95.}	71.9024	0.587734	newly 5 weeks cumul	126.	602.24	{210., 1059.}	385.746	0.650013	newly 66 weeks cumul 757. 704.99 {299., 1265.} 43.4095 0.258434
49	newly 3 weeks spec	30.	43.33	{1., 120.}	118.633	0.639115	newly 3 weeks cumul	64.	310.48	{19., 825.}	409.719	0.62628	newly 5 weeks spec	69.	29.24	{0., 96.}	77.8841	1.36966	newly 5 weeks cumul	166.	372.87	{24., 828.}	168.572	0.529696	newly 68 weeks cumul 950. 485.15 {35., 917.} 49.9211 0.567583
50	newly 3 weeks spec	39.	98.93	{7., 216.}	181.462	0.461674	newly 3 weeks cumul	92.	457.89	{49., 889.}	412.185	0.660405	newly 5 weeks spec	56.	41.05	{2., 88.}	60.6607	0.588097	newly 5 weeks cumul	194.	563.35	{103., 1058.}	206.613	0.47546	newly 66 weeks cumul 717. 656.88 {255., 1227.} 47.696 0.281433
